# Complication Risk Classification in Children and Adolescents With Type 1 Diabetes: Interpretable Machine Learning Study Based on Saudi Clinical Guidelines

**DOI:** 10.2196/81039

**Published:** 2026-05-15

**Authors:** Jalilah Fllatah, Haneen Banjar

**Affiliations:** 1 Department of Computer Science, Faculty of Computing and Information Technology, King Abdulaziz University, P.O. Box 80200, Jeddah, 21589, Saudi Arabia, 966 544027109; 2 Center of Research Excellence in Artificial Intelligence and Data Science, King Abdulaziz University, Jeddah, Saudi Arabia; 3 Institute of Genomic Medicine Sciences, King Abdulaziz University, Jeddah, Saudi Arabia; 4 Centre of Artificial Intelligence in Precision Medicines, King Abdulaziz University, Jeddah, Saudi Arabia

**Keywords:** type 1 diabetes, children and adolescents, complication risk classification, Saudi Diabetes Clinical Practice Guidelines, interpretable machine learning, predictive modeling, P4 medicine, clinical decision support systems, SHAP analysis, Shapley Additive Explanations

## Abstract

**Background:**

Complication risks in children and adolescents with type 1 diabetes (T1D) can lead to serious health outcomes if not detected early. Despite the availability of clinical data, there remains a gap in interpretable tools that support risk stratification in this age group, particularly in alignment with local clinical guidelines.

**Objective:**

The purpose of this study is to develop a clinically interpretable model that classifies the risk levels of T1D complications—acute, chronic, and low—using real-world data and expert clinical rules derived from the Saudi Diabetes Clinical Practice Guidelines.

**Methods:**

A pediatric T1D dataset comprising of 306 patients was preprocessed through structured cleaning and feature engineering. Risk labels were constructed using Saudi Diabetes Clinical Practice Guidelines–derived rules. Feature selection was performed using a hybrid approach that combined the SHAP (Shapley Additive Explanations) analysis with exhaustive feature selection. A decision tree model was trained and optimized via cross-validation, using the *F*_1_-score as the primary performance metric.

**Results:**

The final model achieved a high mean *F*_1_-score of 0.9876 with a low variance of 0.0189, using only 5 clinical features: BMI, hypoglycemia, disease duration, hemoglobin A_1c_, and impaired glucose metabolism. These features were consistently ranked as the most influential. The resulting decision tree offered a transparent logic path, enhancing its clinical interpretability and usability.

**Conclusions:**

This study demonstrates that a simple and interpretable model, guided by national clinical guidelines, can effectively predict the risk levels of T1D complications in children and adolescents. Its strong performance, clarity, and reliance on a small number of clinically meaningful features make it a promising candidate for integration into clinical decision support systems. This supports a shift toward predictive and personalized diabetes care.

## Introduction

### Background

Type 1 diabetes (T1D) is an autoimmune condition in which the body’s immune system selectively destroys pancreatic beta cells, resulting in absolute insulin deficiency and lifelong dependence on exogenous insulin. It primarily affects children and adolescents and requires continuous insulin administration, glucose monitoring, precise dietary management, and lifestyle adjustments [1]. Recent data from the International Diabetes Federation Diabetes Atlas 2025 indicate a significant rise in the global burden of T1D, with an estimate of 30,000 children and youth worldwide at risk of death due to undiagnosed T1D at onset [2]. In 2024, more than 9.5 million people were living with T1D globally, including approximately 1.9 million children and adolescents [2]. Within this context, Saudi Arabia is among the countries most affected globally, with 46,469 children and adolescents reported to be living with T1D in 2024 [2]. This burden is further compounded during adolescence, when hormonal and psychological changes can affect treatment adherence and increase the risk of complications [3,4].

Acute complications, such as diabetic ketoacidosis (DKA) and severe hypoglycemia, are among the leading causes of mortality in this age group if not promptly addressed. In many patients, approximately 30% of first hospital admissions and initial diagnoses of T1D occur due to DKA [1,4]. DKA occurs as a result of absolute insulin deficiency, which increases lipolysis, leading to uncontrolled hyperglycemia, ketone body production, and metabolic acidosis, and may be fatal if not treated promptly [3,4]. Severe hypoglycemia, on the other hand, results from an imbalance between insulin levels and glucose availability, which can cause seizures, loss of consciousness, or sudden deterioration [4]. Both acute complications represent life-threatening emergencies and remain a significant challenge contributing to mortality in children and adolescents with T1D [1].

In addition to acute risks, long-term poorly controlled T1D leads to chronic complications, including nephropathy, chronic kidney disease (CKD), and neuropathy [3]. These complications may begin during adolescence and progress over time, negatively impacting quality of life and increasing health care and economic burdens [3,5]. Clinical evidence underscores that maintaining blood glucose levels near normal, as monitored by hemoglobin A_1c_ (HbA_1c_), significantly reduces the long-term incidence of these adverse outcomes [3,6]. This highlights the importance of early risk prediction and advanced therapeutic approaches to mitigate long-term adverse outcomes [1,5].

The medical literature emphasizes that early intervention in chronic conditions, such as T1D, can lead to significantly better outcomes. Accurate risk prediction supports more timely preventive care, reduces hospital admissions, improves patients’ daily lives, helps health care providers allocate resources effectively, and also strengthens patient and family education [3-5].

Despite the abundance of available clinical data, a noticeable gap remains in the availability of interpretable tools that assist clinicians in reliable and meaningful risk assessment, particularly for pediatric populations [1,5].

### Prior Work

Machine-learning (ML) studies on predicting diabetes complications have made notable advances. Recent reviews indicate significant advancements in ML-based detection, but persistent challenges remain in translating these models into clinical practice, particularly in integrating them into established medical workflows [7]. Most work has focused on the adult population with type 2 diabetes, with limited attention on children and adolescents with T1D. This focus leaves a gap in pediatric T1D care, where comprehensive multicomplication risk assessment remains limited [8].

Jian et al [5] developed predictive models that achieved high accuracy using common algorithms, such as random forests and decision trees. However, their work focused on adults and chronic complications without leveraging clinical guidelines. Similarly, Ravaut et al [9] applied gradient-boosted models to a large administrative dataset to predict both acute and chronic complications, although the binary outcome approach limits its use for nuanced risk stratification.

Eid et al [10] and Subramanian et al [11] focused on acute complications, such as DKA in pediatric patients, but their models were confined to specific outcomes without broader complication profiling or integration of clinical knowledge. Voskergian et al [12] employed synthetic electronic health records (EHRs) to predict multiple complications while not incorporating guideline-based features or ensuring interpretability.

Deep learning techniques, including deep neural networks, convolutional neural networks, and recurrent neural networks, demonstrate high predictive performance across various medical datasets. However, their lack of interpretability often makes them challenging to adopt in clinical practice [13]. By contrast, ML techniques, such as decision trees and logistic regression, are easier to interpret, making them more appropriate for use in pediatric health care contexts [10,14].

Although interpretation tools, such as SHAP (Shapley Additive Explanations), have improved model explainability [9], many existing studies still depend on basic importance scores or filter-based feature selection techniques and seldom contain domain-specific clinical insights [8,15]. This persistent gap highlights the growing need for approaches that provide transparent explanations, as emphasized by Netayawijit et al [16], while also integrating clinical expertise directly into the model’s logic to ensure relevance [8].

To bridge this gap, this study proposes an interpretable model specifically designed to predict T1D complications in children and adolescents. The model is developed using rule-based features extracted directly from the Saudi Diabetes Clinical Practice Guidelines (SDCPG), and it leverages SHAP and exhaustive feature selection (EFS) alongside a decision tree model to achieve interpretable and accurate predictions.

### Study Objective

The primary aim of this study is to develop an interpretable predictive model designed to classify complication risk levels—low, chronic, and acute—among children and adolescents with T1D. By integrating expert clinical rules from the SDCPG with advanced feature selection methods (SHAP and EFS), this study bridges the gap between high predictive accuracy and clinical transparency. Ultimately, the study provides a locally aligned tool for the Saudi health care system that supports the P4 medicine (predictive, preventive, personalized, and participatory medicine) framework by offering proactive, evidence-based decision support for diabetes management.

## Methods

### Overview

**Figure 1.**
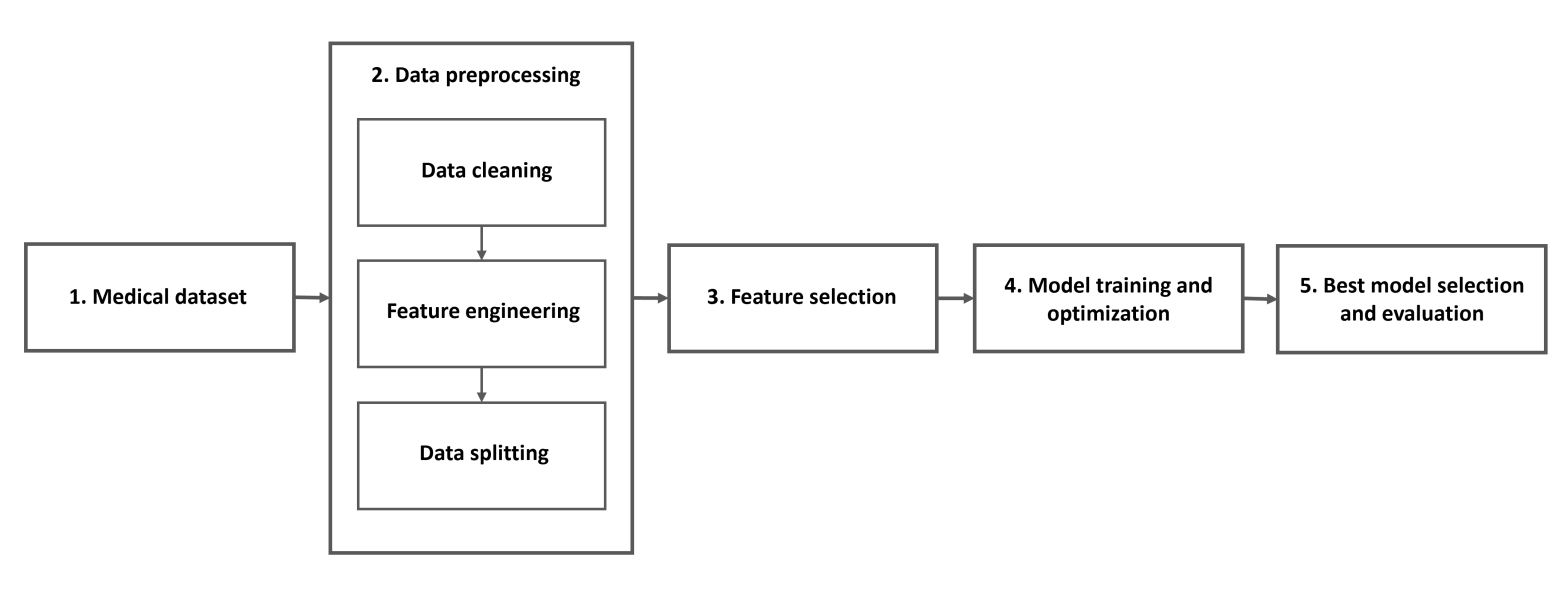
Overall workflow of the multilevel predictive model development for type 1 diabetes (T1D) complication risk classification based on Saudi Diabetes Clinical Practice Guidelines (SDCPG).

The methodology aims to develop a multilevel predictive model that categorizes individuals with T1D based on their risk of complications. This classification is built on rules from the SDCPG. The model classifies patients into 3 main categories: acute risk, denoted by critical conditions, such as hypoglycemia and DKA; chronic risk, involving long-term complications, such as foot deformities, CKD, and neuropathy; and low risk, for patients showing no significant warning signs. By integrating expert clinical knowledge with data-driven modeling, the methodology seeks to identify the most consequential features in this classification. The process is structured into 5 main steps: data collection, data preprocessing, feature selection, training and validating the model, and finally evaluating different models to choose the most accurate one, as shown in Figure 1.

### Dataset

This study utilizes an open-source dataset titled “Dataset on Significant Risk Factors for Type 1 Diabetes,” published in 2018 [17]. The dataset targets children and adolescents in Bangladesh and is designed to explore the major risk factors associated with T1D. It includes 306 participants, with an equal distribution between those diagnosed with T1D and those without, collected through structured surveys from hospitals and diagnostic centers in Dhaka.

The dataset consists of 22 features covering a broad range of information—from demographic characteristics to clinical indicators, such as HbA_1c_ and hypoglycemia, in addition to lifestyle factors, comorbidities, and family history of diabetes. The types of data include categorical, temporal, continuous, and multilabel text variables. Table 1 presents a summary of the dataset features and their corresponding data types. Detailed feature descriptions and categorized values are provided in Multimedia Appendix 1.

**Table 1.** Summary of dataset features and data types used for type 1 diabetes (T1D) risk.

Feature	Type
Age	Categorical
Sex	Binary
Residence	Categorical
HbA_1c_^a^	Binary
Height	Continuous
Weight	Continuous
BMI	Continuous
Disease duration	Temporal
Comorbidities	Multilabel text
Nutrition status	Binary
Mother education	Binary
Growth in infancy	Categorical
Birth weight	Categorical
Autoantibodies	Binary
Impaired glucose metabolism	Binary
Takes insulin	Binary
Insulin delivery	Binary
Family history of T1D	Binary
Family history of T2D^b^	Binary
Hypoglycemia	Binary
Pancreatic affected	Binary
T1D diagnosed	Binary

a HbA_1c_: hemoglobin A_1c_.

b T2D: type 2 diabetes.

### Ethical Considerations

This study involved a secondary analysis of a publicly available and anonymized dataset published by Asaduzzaman et al [17] (2018). The dataset contains deidentified records and does not include any personally identifiable information. As no new data were collected and no direct interaction with human participants occurred, institutional review board approval was not required for this analysis. Informed consent was not required, as the dataset is publicly available and contains no identifiable information. All data were handled in accordance with relevant ethical standards, ensuring the privacy and confidentiality of individuals. No compensation was provided, as this study did not involve direct participant recruitment.

### Data Preprocessing

#### Data Preprocessing Overview

Data preprocessing is the process of preparing raw data by resolving inconsistencies and improving its quality [18]. In this study, it involved 3 main steps. The first step focused on cleaning and normalizing the dataset values. The second step concentrated on engineering relevant features and defining the target variable. The final step included splitting the data into training and testing sets. These steps played an essential role in making the data suitable for ML models.

#### Data Cleaning

Data cleaning is the process used to identify and correct errors and inaccuracies in raw data [18]. In this study, a comprehensive review of missing values within the dataset was conducted. Text formats were cleaned up, and inconsistent labels were fixed to avoid accidental duplicates or misunderstandings when reading the variables. Moreover, column names were standardized to simplify programmatic handling and ensure consistency.

#### Feature Engineering

#### Feature Engineering Overview

Feature engineering is an important stage in data preprocessing, where diverse types of features can affect how accurate the predictive model is [18]. This stage aims to convert cleaned data into numerical formats aligned with the clinical context, improving the model’s ability to detect meaningful patterns and enhance interoperability. In this study, features were categorized into 4 main types: categorical, temporal, continuous, and multilabel text features. Additionally, the dataset did not directly include the T1D risk level as a target variable, so it had to be generated from a set of important clinical variables using rules from the SDCPG. The handling of each feature type is detailed in the following subsections.

##### Categorical Features

Categorical features were converted into numerical values using encoding methods relevant to each variable type. Binary variables, such as “yes” and “no,” were encoded using binary representation as 1 and 0. For multicategory features, such as age, sex, residence, infant growth, birth weight, HbA_1c_ level, and insulin delivery, ordinal encoding was applied in a way that preserved their clinical ordering.

##### Temporal Features

The duration of T1D is considered a key factor in assessing the risk of complications. To ensure consistency, all duration values—originally recorded in days, weeks, months, or years—were converted into a single unit: years, following survival analysis practices outlined by Hosmer et al [19]. To capture the clinical differences in disease duration, values were categorized as short, medium, long, and exceptionally long and were encoded from 0 to 3, as suggested by Dovc et al [20].

##### Continuous Numerical Features

To represent nutritional status, BMI was selected as the primary measure, while height and weight were excluded to minimize redundancy and potential multicollinearity. As the dataset did not include exact age values, BMI was categorized according to the dataset’s age groups based on World Health Organization (WHO) growth standards [21]. These categories—underweight, normal weight, overweight, and obese—were encoded from 0 to 3, as shown in Table S1 in Multimedia Appendix 2.

##### Multilabel Text Features

The comorbidities feature consisted of unstructured free-text entries describing various health conditions. A tailored function was used to clean the data, standardize terminology, and correct duplicate or inaccurate entries. As a result, 23 common comorbidities were extracted, including heart disease, kidney disorders, hypertension, allergies, and others. Each condition was converted into a unique binary feature using a one-hot encoding technique [22], allowing the model to treat them as structured numerical features. This improved the model’s ability to capture associations.

##### Risk Label Construction Based on Clinical Rules

Since the dataset did not include a direct feature indicating the patient’s complication risk level, the target variable was generated by applying a set of clinical if-then rules derived from the SDCPG [23]. These rules were organized into a knowledge base structured around 3 categories: identification, prevention, and management. Only the identification rules were used at this stage to classify patients into 3 risk levels: acute, chronic, and low. The original guideline used for deriving the clinical rules from the SDCPG is publicly available via the Saudi Health Council [24]. Representative clinical identification rules and their corresponding thresholds are summarized in Table 2.

**Table 2.** Representative clinical identification rules derived from the Saudi Diabetes Clinical Practice Guidelines (SDCPG).

Rule ID	Clinical concept	Clinical threshold	Dataset variable(s)	Risk category	SDCPG source
HYPO-01	Hypoglycemia risk associated with insulin therapy	Patient receiving insulin therapy	Takes insulin, and hypoglycemia	Acute	p. 68
CKD-01	Chronic kidney disease risk	T1D^a^ duration ≥5 years	Disease duration	Chronic	p. 82
NEUR-01	Neuropathy risk factors	High BMI or hypertension	BMI and other disease hypertension	Chronic	p. 81

a T1D: type 1 diabetes.

Classification was based on clinical indicators, such as insulin use, hypoglycemia, comorbidities, disease duration, and BMI levels. Because some SDCPG thresholds were not available in the dataset, the rules were implemented using the closest clinical indicators available in the data. To prepare the data for modeling, the labels were encoded as 0 (low), 1 (chronic), and 2 (acute). An initial review of the resulting class distribution revealed a slight imbalance between categories. Although the dataset employed in this study originates from a pediatric population in Bangladesh, the use of rules derived from the SDCPG—grounded in internationally recognized clinical indicators—ensures both consistency and clinical relevance across diverse pediatric T1D populations. Their integration facilitates alignment with evidence-based medical frameworks and provides a standardized approach for risk stratification.

### Data Splitting

The dataset was split into 2 subsets: 80% used for training and 20% for testing. This allowed the model to learn from most of the data and check how well it performs on new, unseen examples. To ensure the class balance remained consistent in both sets, a stratified split was applied. This helped maintain the distribution of the target variable fairly and reduced bias in the results.

### Feature Selection

#### Feature Selection Overview

Following preprocessing and feature engineering, feature selection was used as a key part of the process to reduce the number of input features while optimizing model efficiency and accuracy. The process aimed to identify features that meaningfully contribute to predicting the target variable [24]. This step also helped reduce noise, save computing resources, and enhance clinical interpretability.

Feature selection methods usually fall into 3 groups: filter, wrapper, and embedded. In this study, a hybrid approach was used, starting with an embedded method to reduce the initial list of features, followed by a wrapper method to select the best-performing subset.

#### Embedded Method

Embedded methods work by incorporating feature selection directly into the model training process, allowing the model to evaluate feature importance during training. One popular technique in this category is SHAP. In this study, SHAP was used alongside a random forest model to examine how each input feature contributed to the model’s predictions. Features were then ranked based on their average SHAP values to identify those with the most influence [24].

SHAP offers clinically meaningful insights by showing how each feature contributes to the model’s predictions. Its ability to connect technical outputs with clinical interpretation makes it particularly useful in health care applications. The most influential features selected from SHAP were used in the next step, which helped reduce the number of features, improve computational performance, and simplify the model structure.

#### Wrapper Method

Wrapper methods work by assessing how different feature combinations affect model outcomes and choosing the feature set that achieves the highest evaluation score based on a defined performance metric [24]. In this study, the exhaustive feature selector algorithm was used to explore all combinations of the top 10 most important features identified in the previous step, resulting in a total of 1013 subsets. Each subset was evaluated using a decision tree model and the *F*_1_-weighted score through 5-fold cross-validation (CV). The average performance and standard deviation for each subset were recorded in detail.

Finally, from the 1013 evaluated subsets, the results were filtered to identify the top 5 feature sets based on the highest cross-validated *F*_1_ mean (CV *F*_1_ mean) and the lowest SD (CV *F*_1_ SD). This enabled the selection of combinations that were not only effective and stable but also interpretable from a clinical perspective. These selected subsets were then carried forward for model training and evaluation.

### Model Training and Optimization

#### Model Training and Optimization Overview

After identifying the top 5 feature sets, predictive models were built using a decision tree classifier. The decision tree algorithm was chosen for its simplicity, clarity, and interpretability in clinical environments. The model training process was followed by hyperparameter optimization to enhance generalization and improve predictive performance.

#### Decision Tree Classifier

The classification models in this study were developed using the decision tree classifier from the Scikit-learn library (developed by Pedregosa et al [25]), a widely used ML algorithm known for its ease of interpretation. This method builds a tree-like model by sequentially splitting the dataset based on feature values, creating branches that guide the classification process.

In this work, the Gini impurity criterion was used to determine the quality of each split. This measure captures the degree of impurity in a node and promotes divisions that enhance the separation between different classes [26]. Decision trees are particularly suitable for clinical contexts due to their ability to handle both continuous and categorical features, along with their transparent decision-making logic and interpretable decision paths, which allow health care professionals to trace prediction pathways and support informed decisions [27].

#### Hyperparameter Optimization

To improve model performance and reduce overfitting, hyperparameter tuning was conducted using a randomized search CV. This technique explores a specified number of random combinations within a predefined parameter grid. This tuning technique is designed to efficiently optimize model settings without an exhaustive search [28].

The process focused on adjusting the tree depth, the minimum number of samples required to split a node, and the minimum number of samples at a leaf. Tuning was performed using 10-fold stratified CV and the weighted *F*_1_-score, which balances precision and recall—an important factor for medical data [29]. Tuning was applied to each feature set independently, retaining the best model from each.

### Best Model Selection and Evaluation

To comprehensively evaluate model performance, assessments were conducted in 3 stages: the training set, the test set, and CV to measure consistency and robustness. The evaluation metrics included accuracy, weighted precision, weighted recall, weighted *F*_1_-score, and multiclass area under the curve (AUC). The evaluation focused on average performance and stability, using the mean and SD across folds.

Models were ranked based on their ability to balance performance metrics on the test set, with priority given to strong *F*_1_ and AUC scores combined with low variability. Finally, the decision path of the highest-performing decision tree model was visualized to demonstrate the interpretability of its decision-making process.

## Results

### Target Risk Distribution

The classification of patients into low, chronic, and acute risk categories was guided by clinical rules derived from the SDCPG. The class distribution shows that acute risk constituted the largest group (110/306 patients, 35.9%), followed closely by low risk (101/306 patients, 33%) and chronic risk (95/306 patients, 31%). This fairly balanced distribution enabled model training without requiring specialized class-balancing strategies. The slight increase in acute risk patients is expected, given the age group studied, as children and adolescents are more likely to experience sudden complications, such as hypoglycemia or DKA. In contrast, chronic conditions usually take longer to develop and are less common at younger ages [3].

### Feature Selection Outcomes

#### SHAP Analysis Results

**Figure 2.**
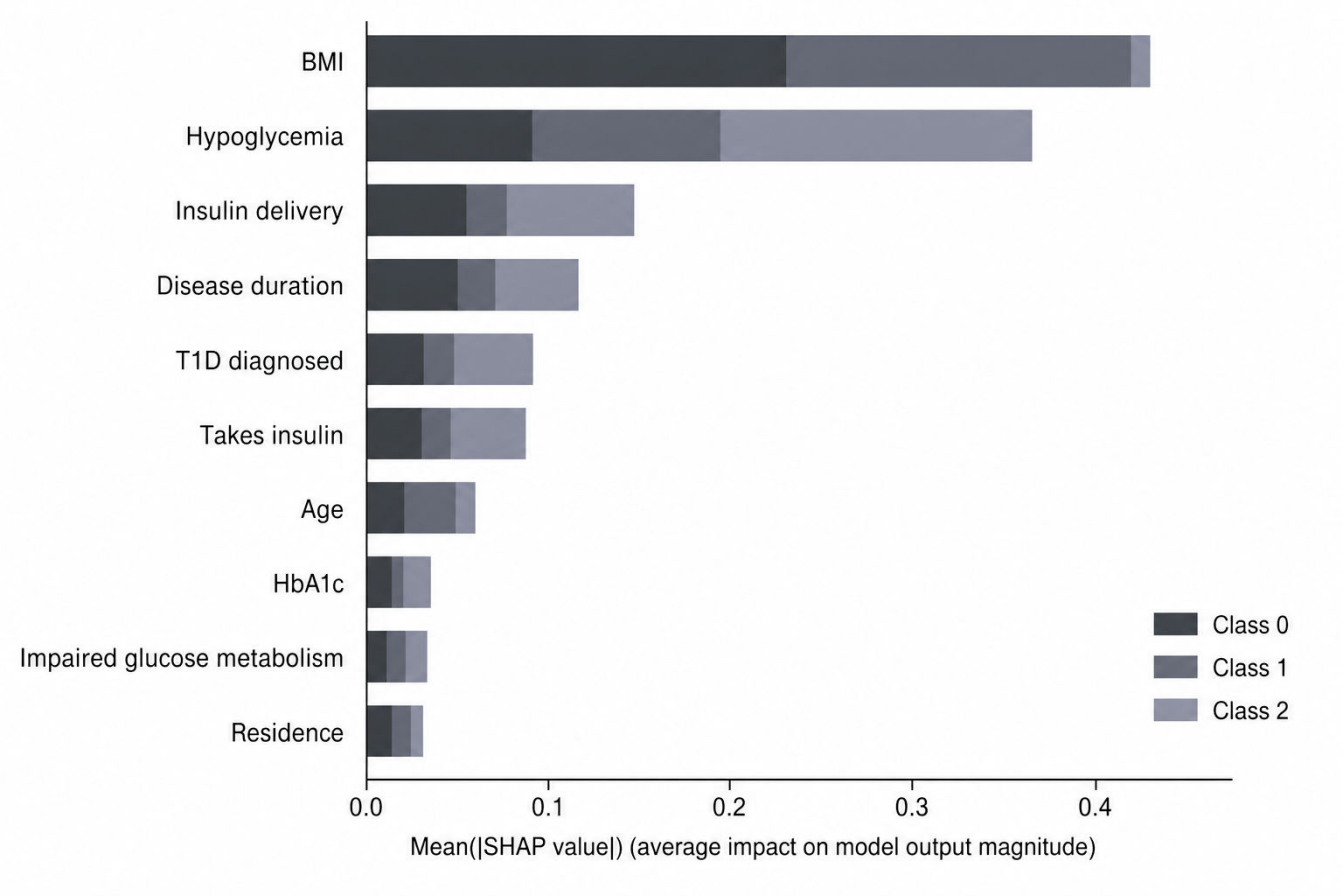
SHAP (Shapley Additive Explanations) summary plot showing the top 10 features ranked by their contribution across all 3 complication risk levels. Feature contributions are displayed using grayscale bars for each class: Class 0=low risk (dark), Class 1=chronic risk (medium), and Class 2=acute risk (light), based on the mean absolute SHAP values. HbA_1c_: hemoglobin A_1c_; T1D: type 1 diabetes.

SHAP was applied to explore how individual features contributed to the model’s predictions. As shown in Figure 2, the SHAP summary plot highlights the top 10 features with the greatest average impact. The results showed that BMI, hypoglycemia, insulin delivery method, and disease duration were the most influential factors in predicting risk levels. BMI emerged as the top contributor, consistent with findings from previous studies that associate obesity with an increased risk of chronic complications, such as cardiovascular disease and hypertension [30-32]. Moreover, hypoglycemic episodes were strongly associated with acute risk, aligning with prior research that identifies such events as among the most dangerous acute complications [33,34]. The insulin delivery method and disease duration showed moderate importance, reflecting their known influence on glycemic control and complication risk reduction [35,36]. Although HbA_1c_, age, and impaired glucose metabolism had relatively lower SHAP values, they remain clinically relevant, particularly HbA_1c_, which is widely recognized as a key indicator of poor glycemic control [37]. The exact SHAP values corresponding to Figure 2 are provided in Multimedia Appendix 3.

#### EFS Subset Results

**Figure 3.**
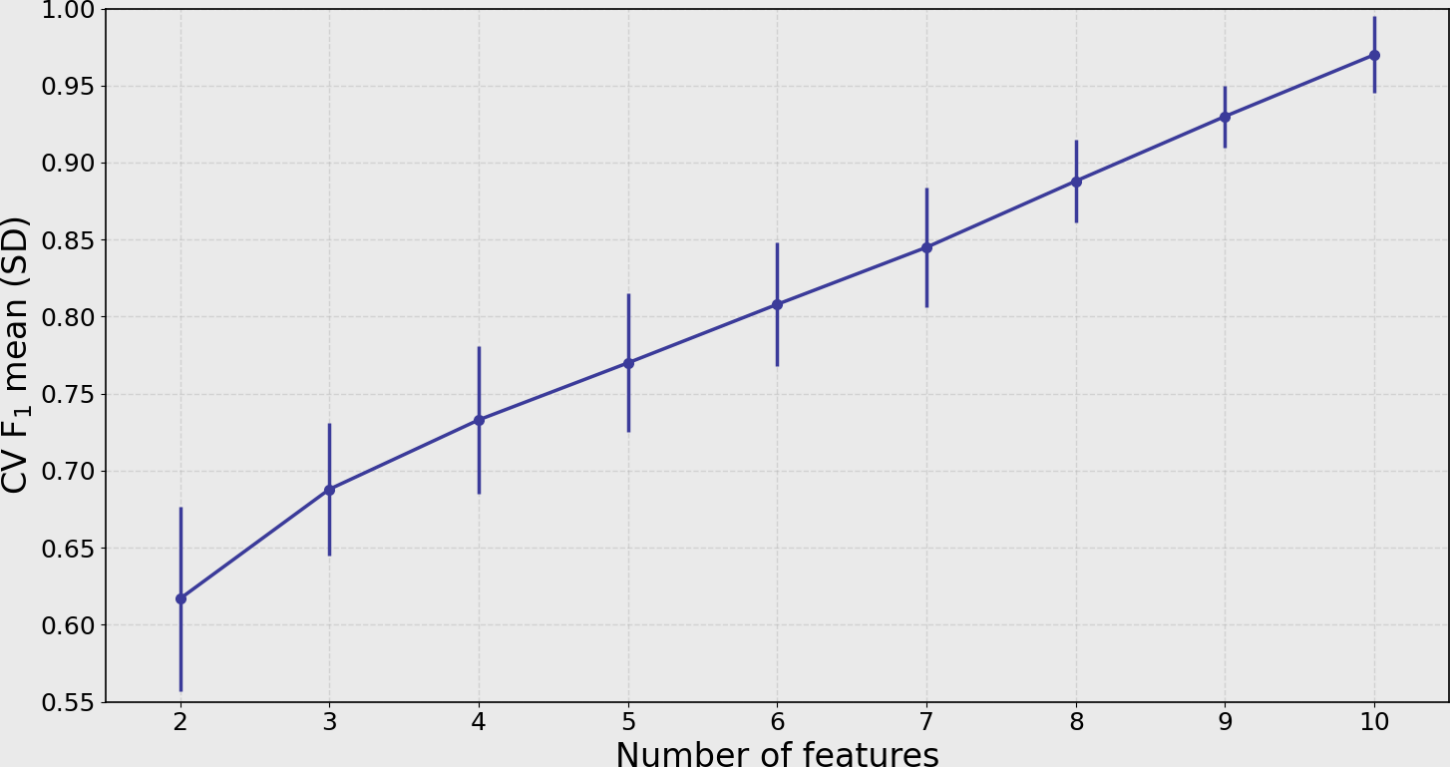
Cross-validation (CV) *F*_1_ performance (mean [SD]) across feature subsets of varying sizes, selected through exhaustive feature selection (EFS) from the top 10 SHAP-ranked features. The plot highlights that feature sets with 5 to 7 features achieve high and stable performance. SHAP: Shapley Additive Explanations.

To build on the SHAP results, an EFS process was applied to identify the best-performing subsets among all possible combinations of the top 10 features. As shown in Figure 3, the relationship between the number of features and model performance shows that classification accuracy (CV *F*_1_ mean [SD]) improved with more features, particularly between 5 and 7 features. Based on these results, the top 5 feature subsets—those with the highest *F*_1_-scores—were selected for full model training and evaluation, as detailed in Table 3.

**Table 3.** Details of the 5 selected feature subsets, which serve as the core input for the training and evaluation phase.

ID	Feature set	Number of features	Train *F*_1_-score	CV^a^ *F*_1_ mean (SD)
C409	BMI + hypoglycemia + disease duration + HbA_1c_^b^ + impaired glucose metabolism	5	0.991832	0.975111 (0.024141)
C640	BMI + hypoglycemia + disease duration + HbA_1c_ + impaired glucose metabolism + insulin delivery	6	0.991832	0.975111 (0.024141)
C670	BMI + hypoglycemia + disease duration +HbA_1c_ + impaired glucose metabolism + T1D diagnosed	6	0.991832	0.975111 (0.024141)
C676	BMI + hypoglycemia + disease duration + HbA_1c_ + impaired glucose metabolism + takes insulin	6	0.991832	0.975111 (0.024141)
C679	BMI + hypoglycemia + disease duration + HbA_1c_ + impaired glucose metabolism + age	6	0.991832	0.975111 (0.024141)

a CV: cross-validation.

b HbA_1c_: hemoglobin A_1c_.

### Model Evaluation Results

Table 4 presents a detailed comparison of 5 predictive models developed using the top-performing feature subsets. Evaluations were conducted across training, testing, and CV phases to assess their accuracy and consistency. All models achieved a test *F*_1_-score of 0.983, with a CV *F*_1_ SD of 0.0189, reflecting strong and consistent performance. Despite performing similarly, all the models shared a common group of 5 core features: BMI, hypoglycemia, disease duration, HbA_1c_, and impaired glucose metabolism. Variations between the models were limited to 1 additional feature per model, but these differences did not result in noticeable performance improvements.

**Table 4.** Comparison of the performance of 5 predictive models based on the best feature sets (SHAP^a^ and EFS^b^).

ID	Feature set	Number of features	Train *F*_1_	Test *F*_1_	CV^c^ *F*_1_ mean (SD)	AUC^d^
1	BMI + hypoglycemia + disease duration + HbA_1c_^e^ + impaired glucose metabolism	5	0.99180	0.98387	0.98761 (0.01892)	0.98661
2	BMI + hypoglycemia + disease duration + HbA_1c_ + impaired glucose metabolism + insulin delivery	6	0.99180	0.98387	0.98761 (0.01892)	0.98661
3	BMI + hypoglycemia + disease duration + HbA_1c_ + impaired glucose metabolism + T1D diagnosed	6	0.99180	0.98387	0.98761 (0.01892)	0.98661
4	BMI + hypoglycemia + disease duration + HbA_1c_ + impaired glucose metabolism + takes insulin	6	0.99180	0.98387	0.98761 (0.01892)	0.98661
5	BMI + hypoglycemia + disease duration + HbA_1c_ + impaired glucose metabolism + age	6	0.99180	0.98387	0.98761 (0.01892)	0.98661

a SHAP: Shapley Additive Explanations.

b EFS: exhaustive feature selection.

c CV: cross-validation.

d AUC: area under the curve.

e HbA_1c_: hemoglobin A_1c_.

The complete evaluation results, including all 1013 feature subsets tested via EFS, and detailed metrics for the top 5 models, are provided in MultimediaAppendices 4  5.

### Final Model Interpretation

**Figure 4.**
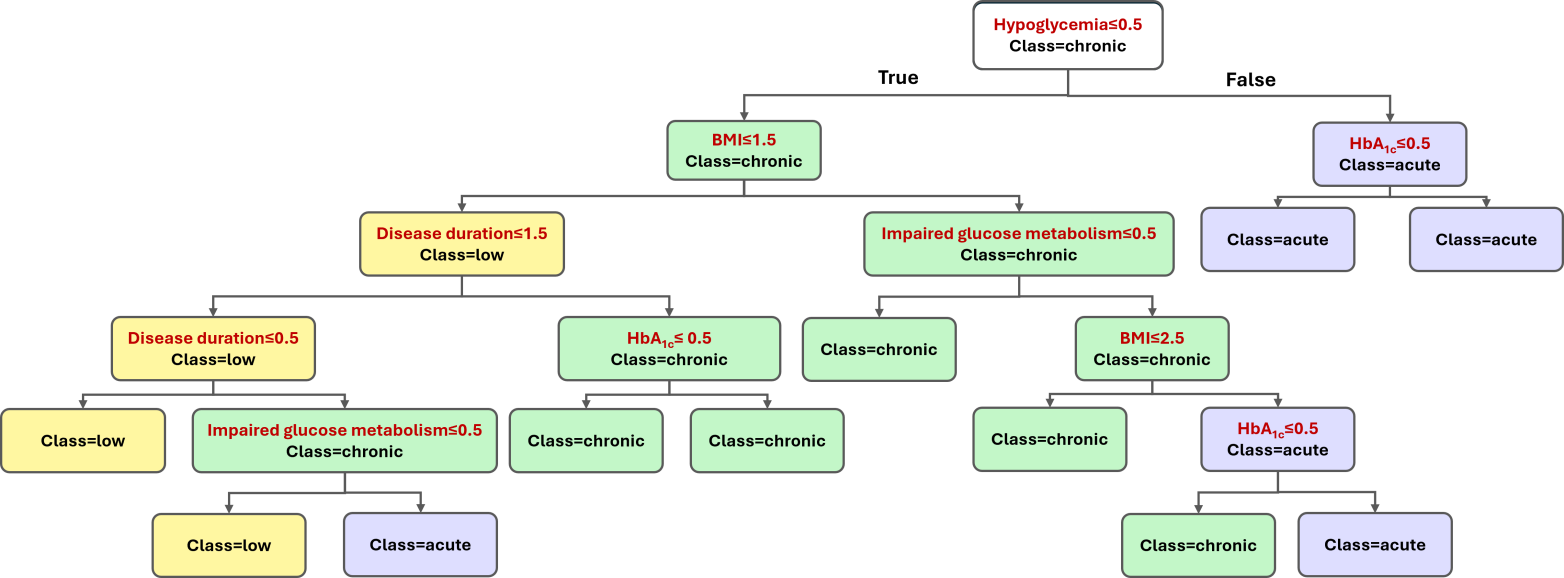
Final decision tree for classifying complication risk levels in children and adolescents with type 1 diabetes (T1D), using 5 clinical features. Risk levels are color-coded as follows: low (yellow), chronic (green), and acute (purple). Each node represents a binary decision, where the left path indicates “true” and the right path indicates “false.”

Although all 5 models demonstrated similar quantitative performance, the first model was selected as the final model due to its structural simplicity. It relies on 5 core features instead of 6 while still maintaining high predictive accuracy. Notably, these 5 features—hypoglycemic episodes, HbA_1c_, BMI, disease duration, and impaired glucose metabolism—were consistently present across all top-performing feature sets. Figure 4 illustrates how the final model makes decisions through a simplified tree that reflects a clinically logical sequence. It begins by checking for hypoglycemic episodes, then evaluates HbA_1c_ if no episodes are recorded. For patients with hypoglycemia, it moves through BMI, disease duration, and impaired glucose metabolism sequentially to reach a final risk classification.

### Illustrative Examples of Risk Classification

To demonstrate how the model processes patient information, Table 5 presents 2 hypothetical patients based on the decision paths shown in Figure 4.

For patient A, the model first checks for hypoglycemia. Because hypoglycemia is present, the model follows the right branch of the tree and evaluates HbA_1c_. Since the HbA_1c_ level is below 7.5%, the patient is classified as an acute risk. For patient B, no history of hypoglycemia is present. The model follows the left branch of the tree and evaluates BMI and metabolic indicators. Because the patient is obese and shows impaired glucose metabolism, the model classifies the patient as a chronic risk.

**Table 5.** Hypothetical patient examples illustrating the decision path of the final decision tree model^a^.

Feature	Patient A	Patient B
Hypoglycemia	Yes (1)	No (0)
BMI	Normal (1)	Obese (3)
Disease duration	Medium (1)	Long (2)
HbA_1c_^b^	Less than 7.5% (0)	Less than 7.5% (0)
Impaired glucose metabolism	Yes (1)	Yes (1)
Predicted risk level	Acute	Chronic

a Values in parentheses represent the encoded numerical values used by the decision tree during preprocessing.

b HbA_1c_: hemoglobin A_1c_.

## Discussion

### Principal Findings

#### Overall Model Performance

This study designed a decision tree model capable of classifying complication risk levels in children and adolescents with T1D using only 5 clinical features. The model achieved a high *F*_1_ mean score of 0.9876 with a low variance of 0.0189, demonstrating both strong predictive accuracy and consistency. These results establish that a small, targeted feature set is adequate to distinguish risk categories effectively, satisfying the study’s goal of creating a simple and interpretable predictive model suitable for clinical integration. Furthermore, the decision tree’s high performance indicates that interpretable models can effectively operationalize the clinical reasoning embedded in the SDCPG.

#### Clinical Interpretation of Selected Features

Importantly, the selected 5 features (BMI, hypoglycemia, disease duration, HbA_1c_, and impaired glucose metabolism) not only achieved high performance but also aligned closely with clinical understanding. The SHAP analysis confirmed that BMI and hypoglycemia were the most impactful predictors: BMI was primarily associated with chronic and low-risk classification, while hypoglycemia strongly influenced acute risk predictions. Disease duration served as a moderate-level contributor that intersected with both chronic and acute risk categories. HbA_1c_ and impaired glucose metabolism had relatively lower SHAP values but were consistently present across all high-performing models, indicating that even features with modest SHAP scores can still contribute meaningfully when viewed in combination.

These selected features are consistent with the SDCPG-derived knowledge base rules summarized in Table 2, which link hypoglycemic events with acute complications, prolonged disease duration, and metabolic risk indicators, such as high BMI with chronic complication risk, including conditions such as CKD and neuropathy. The identification rules explicitly associate insulin therapy with hypoglycemia risk, further supporting the model’s reliance on hypoglycemia as a primary indicator of acute risk. BMI similarly reflects metabolic status indicators outlined in the guideline rules.

In contrast, HbA_1c_ and impaired glucose metabolism emerged as additional metabolic predictors identified through the data-driven feature selection process. While several indicators used during label construction, including BMI, hypoglycemia, and disease duration, remained among the most informative predictors, other variables originally included in the rule definitions, such as hypertension, did not appear among the top-ranked features in the SHAP analysis. Overall, the feature selection process did not produce relationships that contradict SDCPG recommendations; rather, it highlighted the most clinically relevant indicators present in the dataset.

#### Justification for Feature Reduction and Model Transparency

Furthermore, although some of the 6-feature models included additional variables, such as insulin delivery, T1D diagnosis confirmation, or patient age—features that had notable SHAP values—their inclusion did not enhance performance beyond what was achieved with the 5-feature model. Several insulin-related variables, including insulin use (takes insulin) and insulin delivery method, were evaluated during the feature selection process; however, their inclusion did not improve model performance compared with the 5-feature model, as shown in Table 4. This outcome demonstrates that model simplification does not compromise accuracy and highlights that a smaller set of high-quality features can be just as effective. It reinforces the broader principle that feature quality is more critical than quantity and that adding more variables does not necessarily lead to better results. Such simplification enhances the model’s usability in clinical settings, where clarity and transparency are essential. These findings further emphasize SHAP’s strength as an interpretable analytical tool that aligns with clinical reasoning and SDCPG recommendations. Additionally, the EFS process validated the consistency of top-performing feature combinations, reinforcing model robustness and minimizing the risk of overfitting.

### Comparison With Prior Work

To evaluate the contribution of this study, a comparative analysis was conducted with 5 representative ML studies targeting the prediction of diabetes complications. The goal of this comparison is to evaluate how this study differs in methodology, clinical integration, model interpretability, and performance outcomes. Table 6 presents general information on the selected studies, including their primary objectives, targeted complication types, dataset origin and size, and whether they incorporated formal clinical guidelines. Table 7 summarizes the modeling approaches used in each study, reported performance metrics, and levels of interpretability. Including this study, a total of 6 models are summarized.

**Table 6.** General information on compared studies.

ID	Study and year	Purpose	Complication type	Location	Dataset	Based on clinical guidelines
1	Jian et al [5] (2021)	Predict 8 diabetes complications	Chronic	UAE (Ajman)	Structured EHR^a^ (N=884)	No
2	Ravaut et al [9] (2021)	Predict adverse outcomes	Acute and chronic	Canada (Ontario)	Admin health data (>1.5 million)	No
3	Eid et al [10] (2023)	Predict DKA^b^ in pediatric patients	Acute	Saudi Arabia	Structured EHR (N=3737)	No
4	Subramanian et al [11] (2024)	Predict postdiagnosis DKA	Acute	United States (Texas)	Structured EHR (N=1787)	No
5	Voskergian et al [12] (2025)	Predict 4 complications	Chronic	Palestine/Türkiye	Synthetic EHR (~1 million)	No
6	This study (2026)	Classify risk levels in pediatric T1D^c^	Acute and chronic	Bangladesh	Open-source pediatric dataset (N=306)	Yes (SDCPG^d^)

a EHR: electronic health record.

b DKA: diabetic ketoacidosis.

c T1D: type 1 diabetes.

d SDCPG: Saudi Diabetes Clinical Practice Guidelines.

**Table 7.** Summary of modeling approaches and performance in previous work.

Study ID	Model	Performance	Interpretability
1	RF^a^ (*F*_1_=97.7%), SVM^b^ (*F*_1_=96.6%), DT^c^ (*F*_1_=95.2%)	Accuracy: 97.8%	Moderate
2	GBDT^d^	AUC^e^≈77.7	Low-moderate
3	RF (performed best), DT, kNN^f^, GB^g^, AdaBoost, CN2	AUC=0.98, *F*_1_=0.92	Moderate
4	XGBoost^h^	AUC=0.80, *F*_1_=0.78	High (SHAP^i^)
5	XGBoost (AUC=85%), RF (AUC=83%), AdaBoost (AUC=77%), DT (AUC=80%)	Accuracy: 69%‐78%	Moderate
6	DT (5 features), SHAP	AUC≈0.98, *F*_1_=0.98	High (rule-based + SHAP)

a RF: random forest.

b SVM: support vector machine.

c DT: decision tree.

d GBDT: gradient boosted decision tree.

e AUC: area under the curve.

f kNN; k-nearest neighbors.

g GB: gradient boosting.

h XGBoost: extreme gradient boosting.

i SHAP: Shapley Additive Explanations.

A key differentiator of this work is its ability to classify both acute and chronic complication risks through a structured 3-level classification (low, chronic, and acute), whereas most prior studies focused on predicting only a single complication type, typically either acute (eg, DKA) or chronic (eg, CKD). While Ravaut et al [9] addressed both complication types, their model still adopted a binary outcome structure, lacking the nuanced stratification offered in this study. Additionally, this study is the only one among the reviewed works to utilize national clinical guidelines (SDCPG) to inform risk labeling, thereby strengthening its clinical alignment.

Despite using a relatively small open-source dataset (N=306), the proposed model achieved a test *F*_1_-score of 0.98 and AUC ≈ 0.98, on par with or exceeding the performance of more complex models built on larger datasets. Furthermore, it employs only 5 clinically meaningful features and leverages a decision tree classifier enhanced by the SHAP analysis, providing both transparency and clinical explainability. This comparison emphasizes that high predictive accuracy can be achieved without sacrificing interpretability, especially when models are designed with clinical context and usability in mind.

### Clinical Relevance and Usability

The model demonstrated powerful performance while maintaining a clear and interpretable structure, which makes it a practical choice for clinical use. In addition to its technical strengths, this model, based on SDCPG, ensures consistency with local clinical practice. This alignment enhances its credibility and supports smooth integration into existing clinical systems, increasing its potential for real-world adoption. Its ability to identify complication risks in children and adolescents with T1D ensures early intervention and supports a shift toward preventive care rather than reactive treatment.

In this context, the model contributes specifically to the predictive principle of P4 medicine by enabling the early identification of complication risk in this population. While other principles of P4 medicine—such as preventive, personalized, and participatory strategies—require further integration, this model offers a foundational predictive tool to support future enhancements. It not only anticipates complications but also adapts to individual clinical profiles and offers transparent decision paths that can be shared with both patients and health care providers.

### Limitations

While the model yielded encouraging outcomes, several important limitations should be considered. The dataset used in this study was published in 2018 and was derived from a single center, with a relatively small sample of 306 pediatric patients, which may affect the model’s ability to generalize, especially in rare or borderline presentations. Since model validation was conducted without external validation, the findings may not fully translate to real-world settings, which could limit how well the model performs across broader populations.

A further limitation is that the risk labels were generated using SDCPG-derived rules rather than independently observed clinical outcomes, such as confirmed DKA or nephropathy. Therefore, the presented model performance reflects adherence to guideline-based classification logic, rather than prospective prediction of clinical complications. Future longitudinal investigations are required to validate the proposed risk classifications using real-world clinical outcomes.

Additionally, the clinical data were originated from a Bangladeshi cohort, whereas the risk classification rules were derived from the SDCPG. Although this supports guideline portability, differences in health care infrastructure, clinical practices, or population characteristics may influence generalizability. Some clinical features were simplified, such as using age groups instead of exact values, and certain variables were inconsistently recorded or structured, which may reduce predictive precision in pediatric patients. Moreover, the study did not compare its model against an established clinical risk scoring system or clinical decision support system baseline tool, as no standardized benchmark tool was available for this specific population and use case.

Finally, although the model uses common clinical features, such as HbA_1c_, disease duration, and BMI, inconsistencies in data documentation and system integration across health care institutions could pose challenges for its direct implementation into clinical decision support system platforms.

### Future Directions

Future work should aim to validate the model using external datasets from diverse populations to assess its generalizability. Additionally, exploring the integration of other P4 medicine elements, such as personalized treatment pathways and participatory tools, could enhance the model’s utility. Collaboration with health care institutions to embed the model into existing EHR systems and collect feedback from clinicians on usability will be critical for real-world applications and iterative refinement.

### Conclusions

This study developed a clinically meaningful approach to classifying complication risks in children and adolescents with T1D, based on a set of clinical rules extracted from the SDCPG, to build a model that balances accuracy with interpretability. Using a hybrid feature selection technique that combines SHAP and EFS with a decision tree model, the model achieved consistently high performance using only 5 clinical indicators. These results suggest that effective risk classification can be achieved without complex systems, making the model a practical candidate for clinical use. Its transparency makes it easier for health care teams to trust and apply.

Future work should focus on external validation to test its generalizability and on expanding the dataset to improve robustness. Further exploration of the model’s integration within EHR systems and alignment with the broader principles of P4 medicine is also warranted. This study specifically addresses the predictive component, laying the foundation for future work that could incorporate preventive, personalized, and participatory dimensions to enhance diabetes care for children and adolescents.

## Supplementary material

10.2196/81039 Multimedia Appendix 1 Detailed overview of dataset features and categorized values used for type 1 diabetes.

10.2196/81039 Multimedia Appendix 2 BMI classification thresholds used to encode nutritional status across four children and adolescent age groups based on the World Health Organization Growth Standards.

10.2196/81039 Multimedia Appendix 3 Shapley additive explanations values for the top 10 features corresponding to Figure 2.

10.2196/81039 Multimedia Appendix 4 Exhaustive feature subsets (n=1013) with cross-validated *F*_1_ performance metrics.

10.2196/81039 Multimedia Appendix 5 Detailed evaluation metrics for the top 5 predictive models (train/test/CV *F*_1_, area under the curve).

## Abbreviations

AUC: area under the curve
CKD: chronic kidney disease
CV: cross-validation
DKA: diabetic ketoacidosis
EFS: exhaustive feature selection
EHR: electronic health record
*F*_1_: *F*_1_-score (harmonic mean of precision and recall)
HbA_1c_: hemoglobin A_1c_
ML: machine learning
P4 medicine: predictive, preventive, personalized, and participatory medicine
SDCPG: Saudi Diabetes Clinical Practice Guidelines
SHAP: Shapley Additive Explanations
T1D: type 1 diabetes
WHO: World Health Organization
