# Supplementary material for: Complication Risk Classification in Children and Adolescents With Type 1 Diabetes: Interpretable Machine Learning Study Based on Saudi Clinical Guidelines
Source: JMIR Form Res. 2026 May 15;10:e81039. doi: 10.2196/81039 (PMC13178817; doi:10.2196/81039)
Supplement: Multimedia Appendix 1 [file formative-v10-e81039-s001.pdf]

Table 1. Detailed overview of dataset features and categorized values used for T1D risk modeling.

Feature	Description	Type	Categories
Age	Participants are divided into four age groups based on their age at data collection.	Categorical	Greater than 15, less than 15, less than 11, less than 5
Sex	Participants are categorized by their biologically assigned sex.	Binary	Female, male
Residence	Participants are classified by the type of geographic area where they reside.	Categorical	Urban, suburban, rural
HbA1c	Participants' glycated hemoglobin level indicates average blood glucose control.	Binary	Less than 7.5%, over 7.5%
Height	Participants' body height is measured in meters.	Continuous	0.44 – 1.83
Weight	Participants' body weight is measured in kilograms.	Continuous	5 – 87
Body Mass Index (BMI)	BMI is calculated from height and weight as an indicator of weight status.	Continuous	10.01 – 61.98
Disease Duration	Time since diagnosis of T1D is reported in days, months, or years.	Temporal	None, 5 days – 16 years
Comorbidities	The presence of any additional diagnosed medical conditions is recorded.	Multi-Label Text	21 grouped categories[a]
Nutrition Status	Participants' nutritional intake is assessed for adequacy.	Binary	Yes, no
Mother Education	Whether the participant's mother has received formal education is indicated.	Binary	Yes, no
Growth in Infancy	Standardized growth percentile during infancy is recorded based on national references.	Categorical	Lowest, middle, and highest quartiles
Birth Weight	Birth weight percentile is recorded relative to standardized growth charts.	Categorical	Lowest, middle, highest quartiles, and unknown
Autoantibodies	The presence of diabetes-specific autoantibodies is indicated.	Binary	Yes, no
Impaired Glucose Metabolism	Evidence of abnormal glucose regulation before diagnosis is recorded.	Binary	Yes, no
Takes Insulin	The current insulin therapy usage status is recorded.	Binary	Yes, no
Insulin Delivery	The method of insulin administration is recorded.	Binary	Injection, no
Family History of T1D	The presence of T1D in first-degree relatives is indicated.	Binary	Yes, no
Family History of T2D	The presence of T2D in first-degree relatives is indicated.	Binary	Yes, no
Hypoglycemia	Participants' history of hypoglycemia is recorded.	Binary	Yes, no
Pancreatic Affected	Clinical evidence of pancreatic dysfunction or disorders is documented.	Binary	Yes, no
T1D Diagnosed	Participants' diagnosis of T1D is recorded.	Binary	Yes, no

[a] Includes: none, eye disorders, headaches, body or leg pain, fever, skin conditions, thyroid disease, cardiovascular symptoms, allergic responses, kidney issues, liver disease, congenital anomalies, blood pressure problems, thalassemia, seizures, vision loss, hormonal or neurological issues, and others.
