# Supplementary material for: Complication Risk Classification in Children and Adolescents With Type 1 Diabetes: Interpretable Machine Learning Study Based on Saudi Clinical Guidelines
Source: JMIR Form Res. 2026 May 15;10:e81039. doi: 10.2196/81039 (PMC13178817; doi:10.2196/81039)
Supplement: Multimedia Appendix 2 [file formative-v10-e81039-s002.pdf]

Table 1. BMI Classification Thresholds by Age Group Based on WHO Growth Standards

Age Category	Age Range	Underweight	Normal	Overweight	obese
1	Less than 5 years	Below 14	14 to 17	Above 17 to 18.5	Above 18.5
2	Less than 11 years	Below 18	15 to 18	Above 18 to 20	Above 20
3	Less than 15 years	Below 16.5	16.5 to 18	Above 21 to 23	Above 23
4	Greater than 15 years	Below 18.5	18.5 to 24.9	Above 24.9 to 29.9	Above 29.9

References – Multimedia Appendix 2

[1] World Health Organization. WHO Child Growth Standards. Available from: https://www.who.int/tools/child-growth-standards
